# Retropharyngeal calcific tendinitis: A rare, benign, but painful condition with stiff neck

**DOI:** 10.1002/jgf2.360

**Published:** 2020-07-17

**Authors:** Tetsuya Akaishi, Keiki Miura, Masaaki Iduma, Tadashi Ishii

**Affiliations:** ^1^ Department of Education and Support for Regional Medicine Tohoku University Hospital Sendai Japan; ^2^ Department of Orthopedic Surgery Tome City Hospital Tome Japan; ^3^ Department of Internal Medicine Tome City Hospital Tome Japan

**Keywords:** acute neck pain, cervical MRI, retropharyngeal tendinitis

## Abstract

A 51‐year‐old afebrile man visited the hospital with acute severe neck pain with stiff neck, but cervical CT showed no calcification around the odontoid process. Cervical MRI revealed prevertebral hyperintense area of edema at the C1‐C5 vertebral bodies, providing the diagnosis of retropharyngeal calcific tendinitis. The patient was spared for lumbar puncture, and his symptoms swiftly disappeared only with oral NSAIDs.

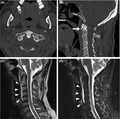

A previously healthy 51‐year‐old man noticed a severe neck pain 3 days before he visited the hospital. He was afebrile, and nausea was absent. However, the neck pain gradually increased, for which he could not move his neck at all. At the first hospital visit, the body temperature was 36.2°C and laboratory testing showed slightly elevated white blood cell count (9790/μL) and CRP level (0.66 mg/dL). Crowned dens syndrome (CDS) was suspected, but cervical spine CT revealed no finding of calcification around odontoid process (Figure 1A). Instead of that, the CT image indicated the presence of calcification anterior to the odontoid process (Figure 1B). Cervical MRI revealed prevertebral hyperintense area of edema at the C1‐C5 vertebral bodies (Figure 1C: T2WI, Figure 1D: STIR), which revealed the diagnosis of retropharyngeal calcific tendinitis (RCT). NSAIDs brought swift remission of symptoms.

**FIGURE 1:**
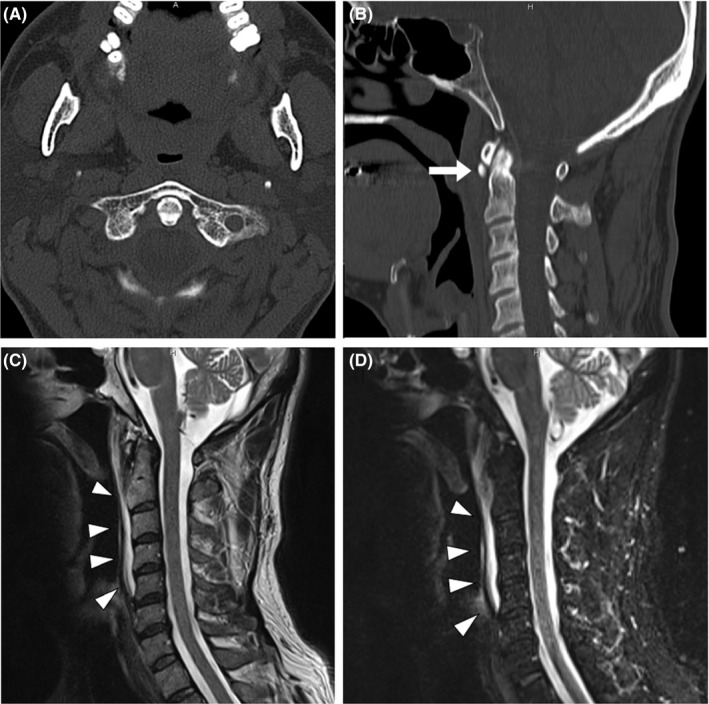
A, Cervical spine CT with horizontal slices in bone window revealed no finding of calcification around odontoid process; B, cervical spine CT with sagittal slices revealed calcification anterior to the odontoid process; C, T2‐weighted image in cervical MRI revealed prevertebral hyperintense area of edema at the C1‐C5 vertebral bodies; D, STIR image in cervical MRI

Retropharyngeal calcific tendinitis is a relatively rare, usually self‐remitting, benign condition caused by the deposition of basic calcium phosphate in the tendons of the longus colli muscle. Retropharyngeal calcific tendinitis usually occurs in younger population than CDS^1^, ^2^. Cervical spine CT and MRI are useful to diagnose these conditions, but head CT can be substituted if the upper cervical spine level is included in the evaluated area. The prevertebral tendinitis mostly locates at the C1‐C2 level, but lower levels (ie, C4‐C6 levels) can be also involved^3^. The patients usually visit hospitals with complaints of severe neck pain and odynophagia, with which they are often misdiagnosed with other serious conditions, such as retropharyngeal abscess or bacterial meningitis^4^. Both of the patients with RCT and CDS can be afebrile only with a slight elevation in the serum CRP level like the present case^5^, although most of the patients are febrile. As a tip of differential diagnosis, most patients with RCT are 30‐60 years old at onset, whereas most patients with CDS are over 60 years old at onset^2^. Although the neck pain in RCT and in CDS is quite severe and usually exhibits neck stiffness, these two conditions are benign conditions that can be symptomatically treated with NSAIDs or acetaminophen. Another point to correctly diagnose these conditions is to evaluate the cervical paraspinal calcification in bone window of the CT scan. Because odontoid process is not usually included in the range of head CT scan, clinicians are recommended to order the cervical CT scan or to specify that the odontoid level to be included in the head CT scan. Correct diagnosis of these two conditions is important to avoid unnecessary invasive diagnostic examinations, such as lumbar puncture or biopsy. A conceivable diagnostic strategy for patients presenting acute severe neck pain with neck stiffness is shown in Figure 2. Avoiding unnecessary invasive examinations with correct differential diagnosis is truly important, but it is more important not to overlook the cases with urgent conditions, such as strokes, subarachnoid hemorrhage, and meningitis.

**FIGURE 2:**
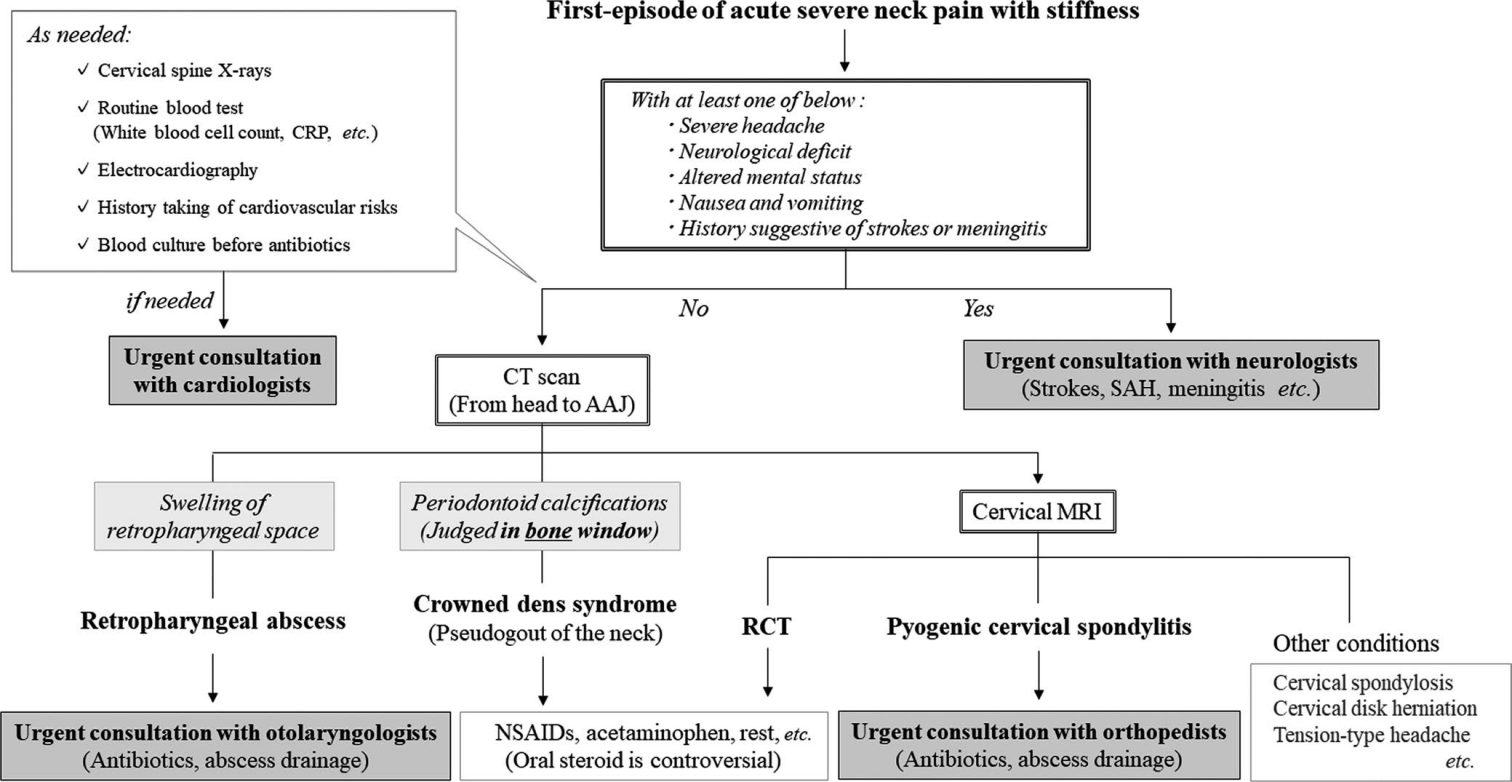
A conceivable diagnostic strategy for patients with acute painful stiff neck is shown. AAJ, atlanto‐axial joint; CRP, C‐reactive protein; NSAIDs, non‐steroidal anti‐inflammatory drugs; RCT, retropharyngeal calcific tendinitis; SAH, subarachnoid hemorrhage

## CONFLICT OF INTEREST

The authors have stated explicitly that there are no conflicts of interest in connection with this article.

## AUTHOR CONTRIBUTION

All the authors made substantial contribution to prepare and critically revise the manuscript.

## INFORMED CONSENT

The patient's informed consent was obtained for this case report.
